# Cryptococcus pneumonia presenting in an immunocompetent host with pulmonary asbestosis: a case report

**DOI:** 10.1186/1752-1947-6-170

**Published:** 2012-06-28

**Authors:** Judah P Guy, Shahzad Raza, Elliot Bondi, Yale Rosen, Dong-Sung Kim, Barbara J Berger

**Affiliations:** 1 Department of Internal Medicine, Brookdale University Hospital & Medical Center, New York, NY, 11212, USA; 2 Department of Pathology, Brookdale University Hospital & Medical Center, New York, NY, 11212, USA

**Keywords:** Cryptococci, Asbestos, Fluconazole, Ferruginous bodies, Mycobacterium tuberculosis

## Abstract

**Introduction:**

Cryptococcal infections pose a diagnostic challenge in an immunocompetent host. Asbestos exposure has been associated with pulmonary aspergillosis. This case highlights an interesting presentation of cryptococcal lung inflammation with underlying asbestosis.

**Case presentation:**

A 63-year-old Mediterranean Caucasian woman presented with progressive dry cough of nine months duration. A computed tomography (CT) scan of her chest revealed multiple foci in the right infra-hilar region, which were seen as hot lung masses on a positron emission tomography (PET) scan. These multiple foci appeared metastatic in nature throughout both lung fields with early mediastinal invasion. A computed tomography (CT)-guided core biopsy was obtained from a dominant right lower lobe lung mass. Histology showed chronic granulomatous inflammation with numerous budding yeast forms that were GMS-, PAS-, and mucin-positive, consistent with cryptococcosis together with asbestos bodies (ferruginous). She was managed with fluconazole (400mg (6mg/kg) per day orally) daily. At her six-month follow up, she had marked improvement in her general condition along with a diminution of the lower lobe lung mass.

**Conclusion:**

We report a clinical and radiological improvement in a patient treated for cryptococcal pneumonia. Asbestos exposure was likely to have been an important pathophysiological precursor to infection by environmental fungi.

## Introduction

The pathogenic role of asbestos exposure prior to the development of pulmonary cryptococcal infections is not well defined. A few clinical reports [1-3] have linked asbestos exposure and pulmonary aspergillosis. None of the case reports with prior exposure to asbestos have been linked to pulmonary cryptoccocal infection. Diseases related to *Cryptococcus neoformans* or *C. gattii* have become increasingly prevalent in immunocompromised patients (including those with AIDS, prolonged treatment with glucocorticoids, organ transplantation or malignancy). There are descriptions of pulmonary cryptococcosis in apparently immunocompetent patients [4,5]. Typically, infection first occurs when the patient inhales the basidiospore form of the fungus. A small focal pneumonitis develops that may or may not be symptomatic. Since certain cryptococcal strains may be more virulent than others, the systemic inflammatory response varies from one patient to another. Cryptococci have a tropism for the central nervous system (CNS); after the CNS, the respiratory system is most commonly affected. The clinical manifestations of pulmonary cryptococcosis include localized nodular lesions, with or without cavitation, segmental pneumonic infiltrates, patchy interstitial or alveolar infiltrates, pleural effusions, hilar masses and thoracic lymphadenopathy. This case highlights an interesting presentation of cryptococcal lung inflammation with underlying asbestosis.

## Case presentation

A 63-year-old Mediterranean Caucasian woman presented with mild dyspnea on exertion and a progressive worsening of a dry cough of nine months’ duration. Born in Turkey, she had a history of exposure to asbestos via building materials commonly used in eastern Turkey. She immigrated to the United States at the age of 37. Shortly after arrival in New York she was diagnosed with and treated for pulmonary tuberculosis. (The diagnosis and treatment records were confirmed by the New York City Department of Health.) She had no fever, night sweats, hemoptysis or weight loss. She had no history of allergies; she had never smoked or used illicit drugs. Other than her birth in Turkey and immigration to the US she had no other travel history.

**Figure 1:**
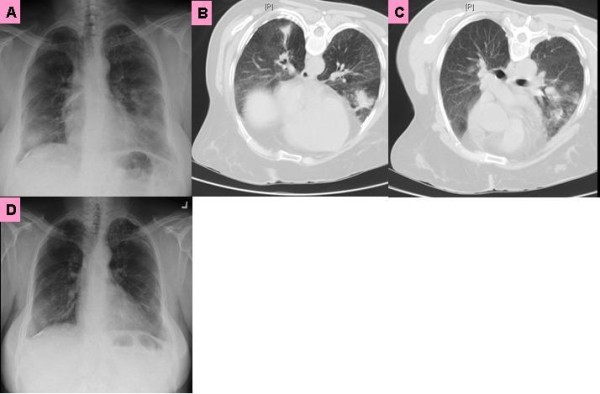
** A. Chest X-ray shows fibrocalcific changes, right lower lobe mass. B** and **C**: Chest computed tomography (CT) scan shows pleural scarring in multiple foci, throughout both lung fields with early mediastinal invasion. Right lower lobe mass (biopsy site). Chest X-ray at four month shows diminished right lower lobe opacity.

**Figure 2:**
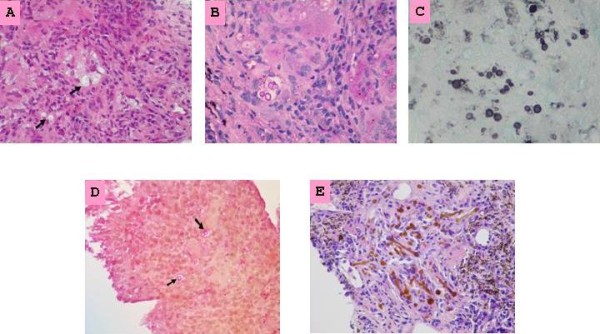
**Cryptococci surrounded by a clear capsule (black arrows) are present in a background of non-specific chronic inflammation with multinucleated giant cells present in the left upper quadrant.** (**A**) Hematoxylin and eosin stain. (**B**) The fungi are clearly seen with a PAS stain. Budding forms are evident. (**C**) The GMS stain demonstrates yeast forms with marked size variation and budding and narrow-based budding, typical features of Cryptococcus. (**D**) The outer wall of the fungi stain positive with the mucicarmine stain and the organisms (black arrows) are surrounded by a clear capsule. A positive mucicarcmine stain confirms the diagnosis of cryptococcosis. (**E**) Multiple ferruginous bodies.

Initial examination included a chest X-ray, which showed fibrocalcific changes at the upper lung fields bilaterally as well as a right lower lobe mass density. A computed tomography (CT) scan of the chest revealed multifocal pleural scarring, appearing metastatic in nature, throughout both lung fields with early mediastinal invasion in the right infrahilar region (Figure 1). Positron emission tomography (PET) scan revealed FDG (fluorodeoxyglucose (^18^ F))-PET positive multiple lung masses. A computed tomography (CT)-guided core biopsy of a dominant right lower lobe lung mass was obtained. The histopathological specimen findings included chronic granulomatous inflammation (multi-nucleated cells with central necrosis) with numerous budding yeast forms that were positive for Grocott's methenamine silver stain (GMS), Periodic acid-Schiff (PAS) and mucin stain, consistent with cryptococcosis. Ferruginous bodies were also present, consistent with asbestos bodies (Figure 2). Cultures of the specimen examined for acid fast bacilli and fungi were negative, as were bacterial cultures. Serum cryptococcal antigen and HIV enzyme-linked immunosorbent assay (ELISA) were negative. A summary of the significant clinical data is illustrated in the table.

She was treated with fluconazole (400mg (6mg/kg) per day orally) for four months. She reported a marked improvement in her symptoms and improvement in her general condition. At her four-month follow up, a chest radiograph showed a significant reduction of the basilar infiltrates bilaterally.

## Discussion

Disease related to *C. neoformans* or *C. gattii* has become increasingly prevalent in immunocompromised patients (including those with AIDS, prolonged treatment with glucocorticoids, organ transplantation or malignancy). There are descriptions of pulmonary cryptococcosis in apparently immunocompetent patients [4,5]. Typically, infection first occurs when the patient inhales the basidiospore form of the fungus. A small focal pneumonitis develops that may or may not be symptomatic. Since certain cryptococcal strains may be more virulent than others, the systemic inflammatory response varies from one patient to another. Cryptococci have a tropism for the CNS; after the CNS, the respiratory system is most commonly affected. The clinical manifestations of pulmonary cryptococcosis include localized nodular lesions, with or without cavitations, segmental pneumonic infiltrates, patchy interstitial or alveolar infiltrates, pleural effusions, hilar masses, and thoracic lymphadenopathy. In our case, the patient had progressive localized disease without any systemic inflammatory response; the initial presentation was indolent. Her radiograph showed nodules and masses. These findings, compatible with a chronic infection of low virulence, simulated the presentation of a malignant lung disease.

Patients who are symptomatic with cryptococcal pneumonia can present with cough, chest pain, increased sputum production, fever, weight loss and hemoptysis. Campbell AU et al. reported 32% of the patients with pulmonary cryptococcosis to be asymptomatic; in these cases pulmonary infection was discovered as an incidental finding [4].

Asymptomatic patients with chest radiographic findings suspicious for malignancy who undergo biopsy are occasionally found to have cryptococcosis. Global ecological studies have found *C. neoformans* in soil samples from around the world in areas frequented by birds, especially pigeons and chickens. More than 50 tree species have yielded *C. gattii* especially from decayed hollows, suggesting a possible ecological niche [6].

Eastern Turkey is a region in which asbestos was mined and used in building materials. There are many caves which are a habitat for pigeons and, most likely, cryptococci. This environment was likely a source of both her asbestos exposure and, perhaps, also her exposure to cryptococci (although New York City is also an endemic area for pigeons and cryptococci.).

In determining whether particular environmental, medical, or behavioral risk factors existed among *C. gattii*, MacDougall L et al. found the following risk factors: age ≥50, current smokers, HIV infection, and invasive cancer [7].

Hammar SP et al. [8] have shown that asbestos may cause localized lesions in the lung that clinically and radiographically are misinterpreted as cancer. Pathological examination of nodules from the lung tissue of patients with prior exposure to asbestos showed intraluminal fibrosis and inflammation of the distal airways, a pattern of change frequently referred to as ‘bronchiolitis obliterans organizing pneumonitis’ (BOOP). That asbestos can potentially produce a localized BOOP-like pulmonary lesion in humans was first suggested in a case report from 1961 [9]. Asbestos bodies were present in association with proinflammatory tissue including alveolar macrophages, occasionally containing asbestos bodies within the cytoplasm.

Hillerdal and Heckscher [2] reported aspergillus infection in four persons with asbestos-related lung changes. They found that degenerative changes of the bronchi can occur in asbestotic lungs and lead to both cylindrical bronchiectasis and fibrotic narrowing. Additional asbestos-induced bronchial changes include ‘broom deformation’, in which asbestos pleurisy leaves considerably thickened and fibrotic pleura. The bronchi beneath such pleural scars will be distorted and bent upwards due to shrinkage of the pleural fibrotic tissue [9-11]. These bronchial changes increase the risk for secondary infection, including, but not limited to, fungal growth. Fungal superinfection might also be due to the malfunction of immunological surveillance among patients exposed to asbestos. This complex effect was detailed by Miller [12]. Macrophages are activated, and among other things, attract various populations of lymphocytes, an increased level of autoantibodies in the blood, as well as overall increased levels of immunoglobulin G (IgG), IgA, and IgM. Recent *in vitro* studies have demonstrated that asbestos acts on peripheral T cells as a super-antigen, causing a depressed function of T-lymphocytes, which might be the reason for the increase in autoantibodies [2]. The hypothesis of malfunction of immunological surveillance is supported by the high incidence of malignancies among patients exposed to asbestos [13].

The hypothesis that pulmonary tuberculosis might be the predisposing factor for pulmonary cryptococcosis in our patient is less likely in the absence of active disease. Segal [14] has found that silica-impaired macrophages are incapable of targeting inhaled conidia, impairing the cellular defense to this microbiological challenge, predisposing the patient with silicosis to increased risk for pulmonary mycosis. Coal mine dust and asbestos fibers are probably less toxic to macrophages than crystalline silica. Therefore, those affected with silicosis are more commonly affected by autoimmune diseases and systemic immunologic complications [1]. Future studies should focus on the association of cryptococcal infection with exposure to asbestos and silica dust.

We initially employed the usual diagnostic tools for pulmonary cryptococcosis which include histology, fungal culture, serum cryptococcal antigen, and radiography. However, if the results are inconclusive, biopsy is recommended. The yeast form of cryptococci can be identified in histopathological specimens using the GMS stain. In our case, budding yeast were identified. Vacuoles in tissue were also seen, likely representing lipid or fat. The mucicarmine stain shows both the yeast form and the capsule and the Fontana-Masson stain reveals melanin contained in the yeast. The identification of *C. neoformans* is supported by the presence of a urease-positive, encapsulated yeast. Further confirmation can be achieved with biochemical tests contained in commercial kits and by detection of the enzyme phenol oxidase, which is solely produced by *C. neoformans.* However, most clinical microbiology laboratories do not stock the expensive agars required for detecting this enzyme. Most laboratories use a variety of sugar fermentations contained in commercial kits to identify the organism.

Fluconazole is active against *C. neoformans*, is easily administered, and has an excellent safety profile. Candidates for treatment clearly include patients with persistent and/or disabling symptoms, those with multiple nodules or extensive infiltrates on chest x-ray, and/or those with positive serum cryptococcal antigen test results.

Additional studies will be needed to determine more precisely the role of fluconazole in the treatment of Isolated pulmonary crptococcosis IPC in immunocompetent patients as well as the optimal dosage and duration of therapy [15]. Similarly, the optimal fluconazole dosage has not been well defined. Dromer et al. [16] and Yamaguchi et al. [17] both employed a daily fluconazole dose of 200 to 400mg, whereas Yew et al*.*[18] utilized an ‘induction’ dose of 600mg for four weeks, followed by 400mg for 10 to 12 weeks. In our case, the patient was treated with fluconazole (400mg (6mg/kg) per day orally). At her four-month follow up, there was marked improvement in her general condition along with diminution of the lower lobe lung mass on her repeat chest X-ray.

## Conclusions

We report an interesting case of cryptococcal pneumonia with successful clinical and radiological improvement with fluconazole. In view of her history of asbestos exposure, antecedent treatment for tuberculosis, lack of systemic inflammatory response and a radiologic appearance of a mass lesion, malignancy was suspected. Biopsy was essential to exclude malignancy. The histological findings of ferruginous bodies confirmed the radiographic appearance of asbestosis. In this case, asbestos exposure was likely to have been an important pathophysiological precursor both to the patient’s tuberculosis and to her susceptibility to infection by environmental fungi.

## Consent

Written informed consent was obtained from the patient for publication of this manuscript and any accompanying images. A copy of the written consent is available for review by the Editor-in-Chief of this journal.

## Competing interests

The authors declare that they have no competing interests.

## Authors’ contributions

JG contributed to analysis of the data, creation of the conceptual design, literature search, interpretation of results, and writing of the manuscript. SR also aided in interpreting the results, searching the literature, acquiring data, and writing the manuscript. EB provided the background and design of the study and a careful review of the manuscript and helped to write the manuscript. YR provided pathological input to the case and reviewed the manuscript. DK provided pathological input to the case and reviewed the manuscript. BB provided background on the study, aided in acquisition of data, and helped with the design and interpretation and supervised the authors. All authors read and approved the final manuscript.
